# Virtual Reality Rehabilitation from Social Cognitive and Motor Learning Theoretical Perspectives in Stroke Population

**DOI:** 10.1155/2014/594540

**Published:** 2014-01-09

**Authors:** Bita Imam, Tal Jarus

**Affiliations:** ^1^Rehabilitation Sciences Graduate Program, University of British Columbia, Vancouver, BC, Canada V6T 2B5; ^2^Department of Occupational Science & Occupational Therapy, University of British Columbia, Vancouver, BC, Canada V6T 2B5

## Abstract

*Objectives.* To identify the virtual reality (VR) interventions used for the lower extremity rehabilitation in stroke population and to explain their underlying training mechanisms using Social Cognitive (SCT) and Motor Learning (MLT) theoretical frameworks. *Methods.* Medline, Embase, Cinahl, and Cochrane databases were searched up to July 11, 2013. Randomized controlled trials that included a VR intervention for lower extremity rehabilitation in stroke population were included. The Physiotherapy Evidence Database (PEDro) scale was used to assess the quality of the included studies. The underlying training mechanisms involved in each VR intervention were explained according to the principles of SCT (vicarious learning, performance accomplishment, and verbal persuasion) and MLT (focus of attention, order and predictability of practice, augmented feedback, and feedback fading). *Results.* Eleven studies were included. PEDro scores varied from 3 to 7/10. All studies but one showed significant improvement in outcomes in favour of the VR group (*P* < 0.05). Ten VR interventions followed the principle of performance accomplishment. All the eleven VR interventions directed subject's attention externally, whereas nine provided training in an unpredictable and variable fashion. *Conclusions.* The results of this review suggest that VR applications used for lower extremity rehabilitation in stroke population predominantly mediate learning through providing a task-oriented and graduated learning under a variable and unpredictable practice.

## 1. Introduction

Stroke is a global, debilitating problem which is increasing both in prevalence and incidence [1, 2]. Stroke ranks as the second highest cause of death and as one of the main causes of acquired adult disability [3, 4]. It is reported that between 55 and 75% of stroke survivors suffer from motor impairments which substantially reduce the quality of their life [5, 6]. Therefore, during rehabilitation, stroke survivors must learn or relearn voluntary control over the affected muscles. The current standard of care for stroke rehabilitation is comprised of physical therapy and occupational therapy that help motor skills learning or relearning after stroke. However the standard rehabilitation for stroke is labour- and resource-intensive, tedious and often results in modest and delayed effects in clients [7, 8]. As a result, the demand for alternative rehabilitation resources has recently become more highlighted [9]. One proposed novel solution is virtual reality (VR) technologies [8, 10, 11]. VR is a computer-human interface that allows users to interact with computer-generated virtual environments (VE) through engaging in different tasks in real time. Promising results have been reported by studies regarding the benefits of VR treatment for motor learning or relearning after stroke [10].

To date, different well-developed theories have been proposed to elucidate the underlying mechanisms involved in maximizing learning. Two key learning theories are Social Cognitive Learning Theory (SCT) and Motor Learning Theory (MLT). Self-efficacy is the keystone of SCT and it is directly linked to learning or acquisition of the target behaviour [12, 13]. Self-efficacy refers to an individual's assessment of his or her capability to perform a particular task. Self-efficacy is enhanced mainly through: vicarious learning, performance accomplishments, and verbal persuasion. Vicarious learning is learning through observing and imitating others' behaviours. Observing others successfully accomplishing certain tasks provides a sense of self-efficacy to the observer that they, too, have the ability to accomplish the task. Imitation takes place most effectively if there is a close identification between the model and the observer. The principle of performance accomplishments is the process of learning through doing the task. Once simple tasks are achieved, more complex tasks are introduced. When improvement in performing a particular task is achieved, the individual will have a sense of mastery or feeling of accomplishment over the task. The acquired sense of mastery will increase self-efficacy. Verbal persuasion is providing encouragement or instruction to the learner while performing a certain task.

MLT is defined as a series of internal processes that lead to relatively permanent changes in the capability to perform certain tasks as a direct result of practice or experience [14]. The processes are broken down into three phases: acquisition, retention, and transfer. The acquisition phase is indicative of the performance level while the retention and transfer phases are indicative of the learning of the task [15]. For instance, in a VR therapy that aims to retrain clients to walk safely, the client would practice how to walk safely in laboratory environment (acquisition), should be able to reproduce the task at a later time (retention), and should be able to walk in the community (transfer) [16, 17]. According to the MLT, the structure of practice, mainly the learner's focus of attention, order and predictability of practice, augmented feedback, and feedback fading, mediates learning [18]. Focus of attention, external focus of attention (i.e., directing attention to the object or to the effect of the action), has been reported to be more effective in enhancing motor learning as compared to internal focus of attention (i.e., directing attention to one's movements) [19–22]. Order and predictability of practice is broken down into predictable/block or invariable and unpredictable/random or variable practice. An invariable practice is repetition of the same activity in a consecutive order (e.g., reaching to pick up the same size, shape, and weight glass for a couple of times in a consecutive order). Variable practice involves performing different activities in an unpredictable, random order (e.g., reaching to pick up different size, shape, and weight glasses in a random order). Unpredictable variable practice is generally more effective than predictable invariable practice in promoting motor learning or retention and transfer [23, 24]. The amount of predictability and variability in practice directly affects learning because it will lead to acquiring the ability to adapt to novel unexpected situations. Augmented feedback involves providing feedback to the learner about their movement patterns or knowledge of performance (KP), as well as feedback about the outcome of the movement or knowledge of result (KR) [14]. For example, corrective feedback given by a therapist regarding improper movement pattern of the learner is a form of KP. The presence of KP and KR is essential to learning because they provide the learner with task-related information about the skill being learned and thereby enhance learning. However, despite the positive effects of augmented feedback, frequent feedback may have negative impact on learning of the task because the learner may make too many corrections during the task that impede performing stable performances when feedback is withdrawn [25]. In addition, too much feedback makes the learner become dependent on an external source of detecting errors, thus preventing the detection of errors independently. Therefore, for optimal learning the frequency of augmented feedback should be reduced or “faded” as the learner's performance improves [25].

The objectives of this systematic review were to (a) identify the VR interventions that have been used for the lower extremity rehabilitation in stroke population and (b) explain their underlying training mechanisms according to the principles of SCT and MLT.

## 2. Method

### 2.1. Search Strategy

The major search terms were VR, stroke, and randomized controlled trial (RCT). Depending on the search engine, subject headings and keywords based around the search terms were used to identify relevant articles. The authors searched the databases Medline (from 1946 to July 11, 2013), Embase (from 1980 to July 11, 2013), and Cochrane Central Register of Controlled Trials (from 2005 to July 11, 2013) via OvidSP. The search terms were adapted for Cinahl (from 1982 to July 11, 2013), which was searched via EBSCO. An example of the Medline search strategy is presented in Table 5. The references of the primary studies were searched for further relevant studies.

### 2.2. Study Selection

Studies published in English were deemed eligible if they met the following criteria.

*(a) Study Design.* RCTs published in peer-reviewed journals.

*(b) Population.* Acute, subacute, and chronic stroke individuals who were 18 years old and older.

*(c) Interventions.* Studies with any form of VR-mediated therapy, including immersive, nonimmersive, and off-the-shelf gaming system technologies.

*(d) Outcomes.* Studies that included at least one validated measure of lower extremity motor function, activity, and recovery.

The two authors independently assessed the studies for inclusion criteria. Any disagreements regarding study selection were documented and resolved in consensus meetings.

### 2.3. Study Quality Assessment

The Physiotherapy Evidence Database (PEDro) scale was employed to assess the quality of the studies that met the inclusion criteria. The PEDro scale is an 11-item scale designed to rate the methodological quality of RCTs [26]. Except for item number 1 which refers to external validity, the rest of the items scored 1 if they are satisfied. Unsatisfied items scored 0. A total score (range = 0–10) is calculated by summing up the individual score of the 10 items. Studies that score lower than 6 are considered low quality [26]. The studies were assessed independently by the two authors and checked against scorings provided in the PEDro website [27]. Any disagreements in quality assessment were resolved in consensus meetings.

### 2.4. Data Extraction

Data extracted included sample, experimental, and control interventions, frequency and duration of the interventions, main outcome measures and data collection timepoints, and main findings.

The VR intervention of each of the selected studies was explained using the SCT and MLT Theories. For the SCT, the VR interventions were assessed to find out if they followed the principles of SCT: vicarious learning (providing the full or partial image of the self or an avatar or a virtual teacher on the screen that could serve as a model), performance accomplishments (presence of graduated learning), and verbal persuasion (provision of instructions or encouragements given during or after the game). For the MLT, the interventions were evaluated to find out whether they followed the principles of MLT's effective learning: learner's external focus of attention, unpredictable and variable practice, and presence of augmented feedback and fading. Checkmarks were used to denote that the VR intervention followed a specific theoretical condition.

## 3. Results

### 3.1. Data Synthesis

**Figure 1:**
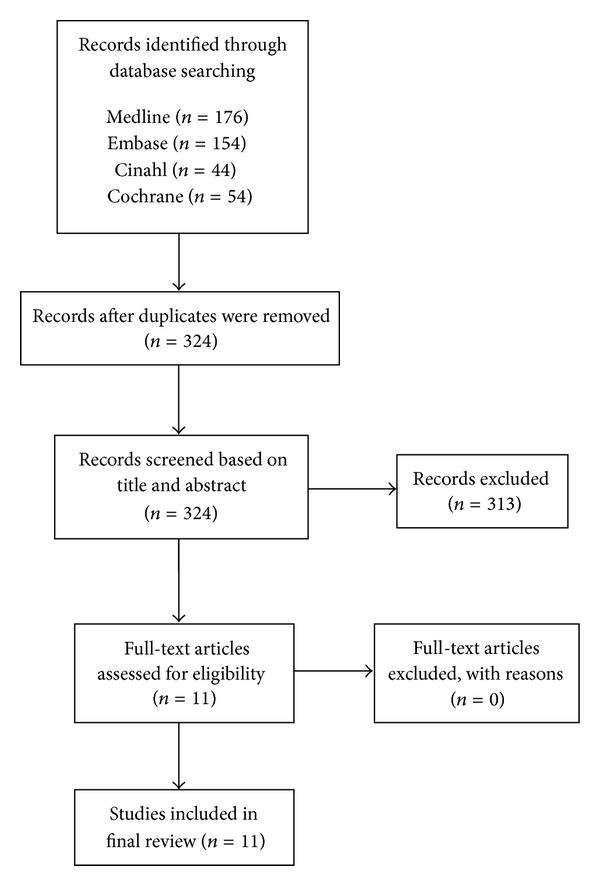
Flow diagram of study selection [39].

Initial search yielded 428 articles. After duplicates were removed, 324 potential articles were identified. The two authors independently evaluated the title and abstract of each of the 324 articles against the study inclusion criteria. From these, 313 articles were excluded based on the title and abstract. Finally, 11 articles were isolated that met the inclusion criteria [28–38]. The details of search result are presented in Figure 1.

### 3.2. Characteristic of Included Studies

Table 1 summarizes the characteristics of the included studies.

*(a) Population. *Subjects in ten studies were in the chronic [28–33, 35–38], whereas in one study they were in the acute phase after stroke [34]. The mean age of the subjects was comparable across studies (from 51.9 to 66.1 years old). None of the studies reported sample size calculation to achieve adequate power to detect clinically important differences. All studies included a small sample size (≤30).

*(b) Interventions. *Different VR applications were used across studies: GestureTek's Interactive Rehabilitation and Exercise System (IREX) [29, 31], VR treadmill training [28, 30, 34, 36, 37], Rutgers Ankle Rehabilitation System [32, 33], and off-the-shelf commercially available gaming systems including Nintendo Wii Fit [35] and Wii Sport, EyeToy Play 2 and Kinect [38]. The frequency and duration of the VR interventions varied across studies from 20- to 60-minute sessions, 3 to 5 times a week for a period of 2 to 6 weeks.

*(c) Outcome Measures. *All eleven studies included more than one outcome measure. Different outcome measures were used to measure ambulation, gait function, and balance. Outcome evaluation was done at baseline and end of treatment in all studies. Five studies included retention outcome evaluation, ranging from 2 weeks to 3 months [28, 30, 32, 33, 38]. All studies but one [38] showed significant improvement in some or all outcomes in favour of the VR group compared to the control group.

### 3.3. Quality Assessment

Table 2 details the quality assessment for each study. The scores ranged from 3 to 7/10. All studies randomly allocated the treatments, although evidence for concealed allocation was unclear in most studies [28, 29, 31–36]. Baseline comparability was achieved in eight studies [28–33, 35, 37], whereas this was unclear in the rest of the studies. Due to the nature of treatments, blinding of subjects and clinicians was impossible. Although Kim et al. [31] stated that subjects and clinicians were blinded, this does not appear possible. Seven studies had a blinded assessor [30–32, 34, 36–38]. Only one study included all randomized subjects in the final analysis (i.e., either no drop-outs or intention-to-treat analysis) [38].

### 3.4. VR Interventions Based on the SCT and MLT

Details of the evaluation of individual VR interventions based on the SCT and MLT are presented in Tables 3 and 4, respectively. Five VR interventions included SCT's vicarious learning by incorporating either the subject's full or partial image (e.g., just the legs) or an avatar of the subject, or a virtual teacher as exercise models in the VE [29, 31, 35, 38]. The principle of performance accomplishments was evident in ten VR interventions by providing graduated learning [28–33, 35–38]. And finally five included verbal persuasion by providing instruction or words of encouragement [32, 33, 35, 37, 38].

Among the MLT's principles, external focus of attention was applied in all eleven VR interventions [28–38]. Nine VR interventions provided unpredictable and variable training [29–35, 37, 38], whereas augmented feedback was presented in seven interventions [28, 29, 31–33, 35, 38]. Feedback fading was provided in only two VR interventions [29, 31].

## 4. Discussion

This was the first systematic review undertaken to attempt to explain the underlying training mechanisms of VR interventions in stroke population based on the SCT and MLT. The SCT and MLT are well-developed theories and have been vastly applied in the design of healthcare interventions [15, 40–42]. To name a few, the concept of SCT has been used in developing effective interventions to increase physical activity adherence in cancer survivors [40] and the elderly [41]. Likewise, the principles of MLT have been used in occupational therapy such as in designing injury prevention programs at work [15] and therapeutic programs for persons with hemiplegia [42].

All studies but one [38] showed significant improvement in outcomes in favour of the VR group compared to the control group. The SCT and MLT might explain the underlying training mechanisms of the VR interventions that resulted in enhanced learning and improvement in the outcomes. The results of this review showed that the SCT's principle of performance accomplishment and MLT's external focus of attention and unpredictable and variable practice were most present in the design of the VR interventions. This suggests that perhaps VR predominantly mediates learning through providing a task-oriented and graduated learning under a variable and unpredictable practice.

Five VR interventions used either a virtual representation of self or an avatar or a virtual teacher as exercise models in different virtual contexts and therefore provided an opportunity for vicarious learning. According to the SCT, people learn by observing and imitating others [12, 13]. The others may be peers, nonpeers, characters, or avatars [43]. The more similar the model to the observer, the greater the degree of imitation and potential for the learning [12, 13]. Therefore, VR interventions that used self-models in the VEs [28, 29, 31] are expected to have provided a higher degree of vicarious learning, thereby enhancing the learning process. This is supported in another study by Fox and Bailenson [44] where they found that the use of virtual representation of self as exercise models was more effective in improving learning than the use of virtual representation of others.

All the VR interventions but one [34] incorporated the principle of performance accomplishment by including a graded form of learning. Once simple tasks were achieved, more complex tasks were introduced by modifying the difficulty of the games. Graded learning allows experiencing incremental success and a sense of accomplishment over the task which ultimately increases self-efficacy and therefore promotes learning [12, 13]. Depending on the virtual scenario, the VR interventions used different strategies to increase the difficulty of the tasks. The difficulty level in Jaffe et al.'s VR intervention was increased by increasing the height and length of the obstacles the subjects had to step over [28]. Other VR systems increased the difficulty of the task by increasing the speed of the games [29–31, 36–38].

Encouragements/instructions were provided through visual and/or auditory stimuli in five VR interventions [32, 33, 35, 37, 38]. For example, the VR intervention in Mirelman 2009 and 2010's studies provided real-time encouragement by a change in the target color from yellow to green along with the word “Great” appearing on the screen after each target was successfully navigated [32, 33]. Providing real-time encouragement increases the motivation and self-efficacy of clients and therefore improves learning [13, 21].

All the VR interventions directed subject's attention externally [28–38]. In other words subject's attention was directed to the effect of the action in the VE, rather than to the motor movements. For example, in the VR intervention in Mirelman 2009, rather than teaching subjects to move their foot in different directions (directing attention to motor movements), subjects learned to navigate a boat in a VE by moving their foot in all directions (directing attention to the object or to the effect of the action) [32]. Since directing subject's attention externally enhances learning [14], this feature of VR training seems to be prominent in mediating learning.

Nine of the VR systems provided training in an unpredictable and variable fashion [29–35, 37, 38]. The amount of unpredictability and variability in a practice directly affects learning because it will lead to acquiring the ability to adapt to novel situations [14]. Since varied practice enhances the ability to adapt to novel situations, it facilitates retention and transfer of the learning to situations where the learner is confronted with novel, unexpected tasks [14]. For example, the VR system in Yang et al.'s study involved avoiding contact with obstacles of different heights and walking in different community scenarios with different speeds on surfaces with different slopes [30]. This provided a richer training in a safe environment because it involved not only walking training but also adapting to various unpredictable scenarios during walking which is more realistic of real-life walking scenarios. Similarly, in You et al.'s study the VR scenario involved capturing stars while avoiding eels and sharks [29]. The eels and sharks were presented in an unpredictable manner and therefore mediated an unpredictable and variable training.

Seven VR interventions provided real-time augmented feedback (KP and KR) in an auditory and/or visual format. Augmented feedback enhances learning through providing the learner with a clear picture of his/her performance [14]. For example, in the VR intervention in Cho et al.'s study, KP was provided by mirroring the learner's movements by showing an avatar on the screen [35]. KR was provided through numerical summaries and auditory stimuli at the end of each game [35]. Although the presence of feedback is important in mediating learning, its frequency needs to be decreased (feedback fading) as the learner improves in the task [14]. Two studies enhanced learning by automatically reducing the frequency of augmented feedback as the subject improved in the games [29, 31]. Feedback fading enhances learning because it prevents the learner from becoming too dependent on an external source of detecting errors, thereby allowing the learner to detect errors independently [25].

## 5. Conclusions

The results of this review showed that the SCT's principle of performance accomplishment and MLT's external focus of attention and unpredictable and variable training were most present in the design of the VR interventions used for lower extremity rehabilitation in stroke population. This suggests that perhaps VR enhances learning predominantly through providing a task-oriented and graduated learning under a variable and unpredictable practice.

## Figures and Tables

**Table 1:** Characteristics of the selected studies (*n* = 11).

Citation	Sample	Experimental/ control intervention	Frequency, duration of intervention	Outcome measure(s)	Data collection	Main findings
Jaffe et al., 2004 [28]	20 chronic stroke patients; mean age = 60.7 years; mean years after stroke = 3.8	*Experimental* VR-based treadmill training (stepping over virtual objects while walking on treadmill) *Control* Stepping over real foam objects on a 10 m walkway	6 sessions of 60 min/session, 3x/week for 2 weeks	Gait velocity and stride length: walking test; balance: balance test; ability to step over obstacles: obstacle test; walking endurance: 6 min talk test.	Baseline, end of treatment, and 2-week retention	Greater improvement in experimental group in gait velocity during the fast walk test (*P* < 0.01). Subjects in both groups improved in stride length, walking endurance, and obstacle clearance capacity.

You et al., 2005 [29]	10 chronic stroke patients; mean age = 57.1 years; months after stroke = 18.8	*Experimental* IREX VR *Control* None	20 sessions of 60 min/session, 5x/week for 4 weeks	Laterality Index (LI) and locomotor recovery: fMRI; motor function: functional ambulation category (FAC) and Modified Motor Assessment Scale (MMAS).	Baseline, end of treatment	Greater improvement in experimental group in the FAC and MMAS (*P* < 0.05). Also, the LI of the SMC area of the VR group increased significantly, compared to the control.

Yang, et al., 2008 [30]	20 chronic stroke patients; mean age = 58.2 years; years after stroke = 6.0	*Experimental* VR-based treadmill training. *Control* Traditional non-VR treadmill training	9 sessions of 20 min/session, 3x/week for 3 weeks	Walking speed: 10 m walk test; community walking time: comfortable pace for 400 m in the community; mobility in ambulatory activities: walking ability questionnaire (WAQ); balance confidence: activities-specific balance confidence.	Baseline, end of treatment, and 1 month retention	Greater improvement in experimental group in the walking speed and community walking time at end of treatment and in WAQ at 1 month retention (*P* < 0.05).

Kim et al., 2009 [31]	24 chronic stroke patients; mean age = 51.9 years	*Experimental* IREX VR *Control* PT	16 sessions of 30 min/session, 4x/week for 4 weeks	Balance: Balance Performance Monitor and Berg Balance Scale (BBS) tests; gait performance: 10 m walk test, Modified Motor Assessment Scale (MMAS), and GAITRite.	Baseline, end of treatment	Greater improvement in experimental group in the BBS, balance and dynamic balance angles, 10 m walk test, velocity, MMAS, cadence, step time, and step length (*P* < 0.05).

Mirelman et al., 2009 [32]	18 chronic stroke patients; mean age = 61.4 years; months after stroke = 48.8	*Experimental* Robotic device + VR (Rutgers Ankle Rehabilitation System) *Control* non-VR robotic device	12 sessions of 60 min/session, 3x/week for 4 weeks	Gait speed: walking on a 7-meter walkway; walking capacity: 6 min walk test; home and community walking: Patient Activity Monitor.	Baseline, end of treatment, and 3-month retention	Greater improvement in experimental group in velocity and distance walked in the lab and in the distance walked and number of steps taken in the community (*P* < 0.05).

Mirelman et al., 2010 [33]	18 chronic stroke patients; mean age = 61.4 years; months after stroke = 48.8	*Experimental* Rutgers Ankle Rehabilitation System *Control* Non-VR robotic device	12 sessions of 60 min/session, 3x/week for 4 weeks	Kinematic (ROM of ankle and hip joints during gait cycle and ROM of the knee joint during stance and swing phases); kinetic of ankle, knee, and hip joints during stance and swing phases of gait; bilateral spatiotemporal parameters (self-selected walking speed, joint kinetics/kinematics).	Baseline, end of treatment, and 3-month retention	Larger increase in experimental group in ankle power generation at push-off (*P* < 0.05) and larger change in ankle ROM (19.5%). Also, greater increase in knee ROM of the affected side of the experimental group during stance and swing.

Yang et al., 2011 [34]	14 acute stroke patients; mean age = 61 years; months after stroke = 16.7.	*Experimental* VR-based treadmill training *Control* Traditional non-VR treadmill training	9 sessions of 20 min/session, 3x/week for 3 weeks	Gait patterns including centre of pressure (COP) related outcomes: during quiet standing, sit-to-stand transfer, and level walking on a 5-meter walkway.	Baseline, end of treatment	Greater improvement in experimental group in COP maximum sway in medial-lateral direction during quiet stance (*P* < 0.05).

Cho et al., 2012 [35]	24 chronic stroke patients; mean age = 64.2 years; months after stroke = 12.6	*Experimental* Nintendo Wii Fit + PT/OT *Control* PT/OT	9 sessions of 30 min/session, 3x/week for 6 weeks	Static balance: force platform. Dynamic balance: Balance Berg Scale, TUG.	Baseline, end of treatment	Greater improvement in BBS and TUG in experimental group (*P* < 0.05).

Jung et al., 2012 [36]	21 chronic stroke patients; mean age = 62 years; months after stroke = 14	*Experimental* VR-based treadmill *Control* traditional non-VR treadmill	15 sessions of 30 min/day, 5x/week for 3 weeks	Dynamic balance: TUG; balance self-efficacy: activities-specific balance confidence.	Baseline, end of treatment	Greater improvement in balance and self-efficacy in experimental group (*P* < 0.05).

Cho and Lee, 2013 [37]	14 chronic stroke chronic patients; mean age = 64.9 years; months after stroke = 78.6	*Experimental* VR-based treadmill using real-world video recording *Control* traditional non-VR treadmill training	18 sessions of 30 min/day, 3x/week for 6 weeks	Balance: Berg Balance Scale (BBS) and TUG; gait analysis (velocity, cadence, paretic side step length, stride length, and single-limb support period).	Baseline, end of treatment	Greater improvement in BBS, TUG, velocity, and cadence in experimental group (*P* < 0.05).

Fritz et al., 2013 [38]	30 chronic stroke patients; mean age = 66.1 years; years after stroke = 3.0	*Experimental* Nintendo Wii Fit and Wii Sport, EyeToy Play 2 and Kinect *Control* normal daily activity	20 sessions of 50–60 min/day, 4x/week for 5 weeks	Lower extremity movement: Fugl-Meyer; balance: Berg Balance Scale; gait and walking: Dynamic Gait Index, TUG, 6 min walk test, and 3-meter walk; and perception of recovery.	Baseline, end of treatment, and 3-month retention	No significant between group differences.

**Table 2:** Quality assessment of selected studies using the Physiotherapy Evidence Database Scale [26].

	Jaffe et al., 2004 [28]	You et al., 2005 [29]	Yang et al., 2008 [30]	Kim et al., 2009 [31]	Mirelman et al., 2009 [32]	Mirelman et al., 2010 [33]	Yang et al., 2011 [34]	Cho et al., 2012 [35]	Jung et al., 2012 [36]	Cho and Lee, 2013 [37]	Fritz et al., 2013 [38]
Random allocation	1	1	1	1	1	1	1	1	1	1	1
Concealed allocation	0	0	1	0	0	0	0	0	0	1	1
Baseline comparability	1	1	1	1	1	1	0	1	0	1	0
Subject blinded	0	0	0	0	0	0	0	0	0	0	0
Clinician blinded	0	0	0	0	0	0	0	0	0	0	0
Assessor blinded	0	0	1	1	1	0	1	0	1	1	1
Data for at least 1 outcome from >85% of subjects	1	1	0	0	0	0	0	1	0	1	1
No missing data or if missing, intention-to-treat analysis	0	0	0	0	0	0	0	0	0	0	1
Between group analysis	0	1	1	1	1	0	1	1	1	1	1
Point estimates and variability	1	1	1	1	1	1	1	1	1	1	0

Total score (/10)	4	5	6	5	5	3	4	5	4	7	6

1 = yes; 0 = no.

**Table 3:** Analysis of studies based on Social Cognitive Theory.

Citation	VR description	Vicarious learning	Performance accomplishments	Verbal persuasion
		*✓*: full or partial self-representation or avatar or virtual teacher (modeling)	*✓*: graduated learning	*✓*: instructions or encouragements during/after the game

Jaffe et al., 2004 [28]	VR-based treadmill: step over 10 identical virtual obstacles while walking on a treadmill. Subjects could see the lateral view of their legs in the VE.	*✓* Partial self-representation	*✓* Subjects were progressed to step over obstacles with larger height and length (harder task) as they improved in the previous session.	x Unclear if gaming system provided any feedback.

You et al., 2005 [29]	IREX VR: the user itself is placed in the VE where they can interact with virtual objects.	*✓* Full self-representation	*✓* Subjects progressed to challenging tasks (speeding up the games or increasing the resistive force) as they improved.	x Unclear if gaming system provided any feedback.

Yang, et al., 2008 [30]	VR treadmill: virtual scenarios at a typical community, including lane walking, street crossing, obstacles striding across, and park stroll. The treadmill's incline and speed alter in conjunction with scenery changes.	x No self-representation or avatar or virtual teacher	*✓* The difficulty of the exercise was progressively increased by increasing the speed of the treadmill, the variety in obstacle heights and surface slopes, and decision making opportunities to avoid collisions.	x Unclear if gaming system provided any feedback.

Kim et al., 2009 [31]	IREX VR: the user itself is placed in the VE where they can interact with virtual objects.	*✓* Full self-representation	*✓* The games progressively became more challenging (speeding up the games or increasing the resistive force) as the subject improved.	x Unclear if gaming system provided any feedback.

Mirelman et al., 2009 [32]	Rutgers Ankle Rehabilitation System: subjects navigated a boat/plane and avoided making contact with a series of targets by moving their foot in different directions.	x No self-representation or avatar or virtual teacher	*✓* Training intensity was based on and progressed according to subject's performance.	*✓* Encouragement was given by the VR after each target was successfully navigated.

Mirelman et al., 2010 [33]	Rutgers Ankle Rehabilitation System: subjects navigated a boat/plane and avoided making contact with a series of targets by moving their foot in different directions.	x No self-representation or avatar or virtual teacher	*✓* Training intensity was based on and progressed according to subject's performance.	*✓* Encouragement was given after each target was successfully navigated.

Yang et al., 2011 [34]	VR treadmill training: virtual walking in a park along a pathway with right/left turns and home activities (turning a light on/off and opening the door).	x No self-representation or avatar or virtual teacher	x Unclear whether graduated learning was involved.	x Unclear if gaming system provided any feedback.

Cho et al., 2012 [35]	Nintendo Wii Fit: subjects stood on a balance board and participated in VEs.	*✓* Subject's avatar	*✓* Subjects were encouraged to increase the difficulty of the games as they improved.	*✓* Encouragement was provided by the VR system after successful score.

Jung et al., 2012 [36]	VR treadmill which immersed subjects in a virtual park stroll.	x Unclear if self-representation or avatar or virtual teacher	*✓* Difficulty gradually increased (increasing the speed of treadmill) as subjects improved.	x Unclear if gaming system provided any feedback.

Cho and Lee, 2013 [37]	VR treadmill using real-world video recording.	x Unclear if self-representation or avatar or virtual teacher	*✓* Difficulty gradually increased (increasing the speed of treadmill) as subjects improved.	*✓* Auditory feedback was provided. Unclear if encouragement was given.

Fritz et al., 2013 [38]	Nintendo Wii Sports and Wii Fit and EyeToy Play 2 and Kinect.	*✓* Subject's avatar and virtual teacher	*✓* Difficulty gradually increased (increasing the speed of treadmill) as subjects improved.	*✓* Encouragements and instructions were provided by the VR system.

Total number of *✓*		5	10	5

**Table 4:** Analysis of studies based on Motor Learning Theory.

Citation	Focus of attention	Order and predictability of practice	Augmented feedback (KP and KR)	KP and KR Feedback fading
	*✓*: external focus of attention	*✓*: random/unpredictable and variable intervention	*✓*: KP and KR were provided	*✓*: feedback fading was present

Jaffe et al., 2004 [28]	*✓* VR directed subject's attention externally.	x Unpredictable but invariable. Subjects stepped over 10 identical obstacles in each trial for 12 times. The speed of the treadmill remained unchanged.	*✓* KP: A tonal sound was provided by the gaming system when subject's foot collided with the obstacle. KR: the computer provided total number of steps and collisions at the end of each trial.	x No feedback fading.

You et al., 2005 [29]	*✓* VR directed subject's attention externally.	*✓* Unpredictable and variable. Three games were practiced. The nature of the games (capturing stars while avoiding eels and sharks) and snowboarding games seem to provide learning in an unpredictable, random VE.	*✓* A high frequency (>90%) auditory and visual KP and KR were provided by the gaming system.	*✓* Frequency of KP and KR was gradually decreased as the performance improved.

Yang, et al., 2008 [30]	*✓* VR directed subject's attention externally.	*✓* Unpredictable and variable. Subjects had to avoid contact with obstacles of different heights, walk with different speeds on surfaces with different slopes. Various unpredictable typical community scenarios: lane walking, street crossing, and obstacles striding.	x Unclear if KP and KR were provided by the gaming system.	x No feedback fading.

Kim et al., 2009 [31]	*✓* VR directed subject's attention externally.	*✓* Unpredictable and variable. Three games were practiced. The nature of the games (capturing stars while avoiding eels and sharks) and snowboarding games seem to provide learning in an unpredictable, random VE.	*✓* A high frequency (>90%) auditory and visual KP and KR were provided by the gaming system.	*✓* Frequency of KP and KR gradually decreased as the performance improved.

Mirelman et al., 2009 [32]	*✓* VR directed subject's attention externally.	*✓* Unpredictable and variable. The position and timing of the targets were altered in an unpredictable, random manner.	*✓* KP and KR were provided by the gaming system.	x No feedback fading.

Mirelman et al., 2010 [33]	*✓* VR directed subject's attention externally.	*✓* Unpredictable and variable. The position and timing of the targets were altered in an unpredictable, random manner.	*✓* KP and KR were provided by the gaming system.	x No feedback fading.

Yang et al., 2011 [34]	*✓* VR directed subject's attention externally.	*✓* Unpredictable and variable. The park scenery had 16 turns (right and left turns) and home activities (such as opening the door and turning the light).	x Unclear if KP and KR were provided by the gaming system.	x No feedback fading.

Cho et al., 2012 [35]	*✓* VR directed subject's attention externally.	*✓* Unpredictable and variable. Six different games with unpredictable nature were used.	*✓* KP and KR were provided by the gaming system.	x No feedback fading.

Jung et al., 2012 [36]	*✓* VR directed subject's attention externally.	x Predictable and invariable. The virtual scenario involved walking in a park with no changes to the VE.	x Unclear if KP and KR were provided by the gaming system.	x No feedback fading.

Cho and Lee, 2013 [37]	*✓* VR directed subject's attention externally.	*✓* Unpredictable and variable. Six virtual scenarios used a sunny or rainy walking track, a walking track with obstacles, daytime or nighttime walking tracks, and walking on trails.	x Unclear if KP and KR were provided by the gaming system.	x No feedback fading.

Fritz et al., 2013 [38]	*✓* VR directed subject's attention externally.	*✓* Unpredictable and variable. Different games were used from Wii Fit, Wii Sport, and EyeToy play 2 and Kinect.	*✓* KP and KR were provided by the gaming system.	x No feedback fading.

Total number of *✓*	11	9	7	2

**Table 5:** Example of Medline search via Ovid.

Term	MeSH	Keywords
Virtual reality	(i) User-computer interface (ii) Video games (iii) Computer simulation	(i) User-computer interface* (ii) Computer simulation (iii) Virtual reality (iv) Computer* model* (v) Video game*

Stroke	(i) Stroke or brain infarction/or brain stem infarctions/or lateral medullary syndrome/or cerebral infarction/or dementia, multi-infarct/or infarction, anterior cerebral artery/or infarction, middle cerebral artery/or infarction, posterior cerebral artery/or stroke, lacunar (ii) Cerebrovascular disorders/or basal ganglia cerebrovascular disease/or basal ganglia hemorrhage/or putaminal hemorrhage/or brain ischemia/or brain infarction/or brain stem infarctions/or lateral medullary syndrome/or cerebral infarction/or dementia, multi-infarct/or infarction, anterior cerebral artery/or infarction, middle cerebral artery/or infarction, posterior cerebral artery/or hypoxia-ischemia, brain/or ischemic attack, transient/or vertebrobasilar insufficiency/or subclavian steal syndrome/or stroke/or stroke, lacunar/ (iii) Hemiplegia	(i) Stroke (ii) Apoplexy (iii) Cva* (iv) Hemipleg* (v) Hemiparesis (vi) Hemiparalysis (vii) (Cerebrovascular or cerebral) adj2 (stroke* or accident*) (viii) Brain infarct*

Randomized controlled trial	Random allocation	Random*
